# Measurement of Fusion Control with Eye Tracking Device in Intermittent Exotropia

**DOI:** 10.3390/diagnostics15030361

**Published:** 2025-02-04

**Authors:** Dong Hyun Kim, Hee Kyung Yang, Sang Beom Han, Jeong Min Hwang

**Affiliations:** 1 Department of Ophthalmology, Seoul National University College of Medicine, Seoul National University Bundang Hospital, Seongnam 13620, Republic of Korea; himrdh@gmail.com (D.H.K.); nan282@snu.ac.kr (H.K.Y.); 2 Saevit Eye Hospital, Goyang 10447, Republic of Korea

**Keywords:** intermittent exotropia, eye tracking device, fusion control

## Abstract

**Background/Objectives**: We wished to develop an automated method for quantifying fusion control in patients with intermittent exotropia (IXT) using an eye tracking device. **Methods**: Fifty subjects fixated on visual targets on an LCD monitor at a distance of 45 cm, consisting of dots moving horizontally and vertically and randomly appearing dots at fixed positions. The control group consisted of participants with less than 5 prism diopters (PD), and the IXT group consisted of IXT patients with 10 PD or more, excluding divergence excess types. Fixation disparity (FD) was measured using an eye tracking device, and the FD score was compared with the Newcastle Control Score (NCS) and the Mayo Clinic office-based scale (OCS) score. The subjects repeated the test twice, and the test–retest reliability was determined. **Results**: The fixation disparity scores of the IXT group during horizontal pursuit, vertical pursuit, and random dot fixation showed positive correlations with the NCS (r = 0.549, 0.583, and 0.481, respectively) and OCS score (r = 0.551, 0.570, and 0.505, respectively). The test–retest reliability of the FD scores of the IXT group using an eye tracking device was fair to good for each task (ICC = 0.633, 0.656, and 0.697, respectively). **Conclusions**: The eye tracking device developed for automated measurement of fusion control has the potential to assist in functional assessments of IXT.

## 1. Introduction

Intermittent exotropia (IXT) is a strabismus condition with outward drifting of either eye interspersed with periods of good alignment or orthotropia [1]. While there is still debate as to whether the natural course of intermittent exotropia continues to worsen, it is clear that neglecting worsening intermittent exotropia leads to visual, functional, cosmetic, and socio-psychological disadvantages [2,3,4]. There are several metrics for assessing worsening intermittent exotropia, including the angle of deviation, stereopsis, symptom scores, and the control score [5,6,7,8,9]. The control score, which assesses the frequency of fusional breakdown, is one of the most important [10]. Deterioration of fusion control, the ability of a patient to maintain orthotropia and control the deviation of their eyes, is considered an indicator for surgical intervention [10].

Currently, scoring systems such as the Newcastle Control Score (NCS) [11,12], the Mayo Clinic office-based scale (OCS) [13,14,15], and Look And Cover then Ten seconds of Observation Scale for Exotropia (LACTOSE) [16,17] are used to measure the degree of fusion control in IXT. When administered by professional specialists, these conventional scoring systems are relatively straightforward and practical for evaluating the progression of intermittent exotropia [13]. However, they have inherent limitations, as they require skilled techniques, which can result in inconsistent accuracy in less experienced examiners. Additionally, subjective scoring by family members can be influenced by the family’s tendency to either exaggerate or undervalue symptoms.

In this study, we developed an automated digital measurement system using an eye tracking device that quantitatively evaluates the degree of fusion control in IXT during various visual tasks. We evaluated the validity and reliability of this new automated measurement system for measuring fusion control in IXT.

## 2. Materials and Methods

### 2.1. Subjects

A total of 50 participants were recruited, who were either invited by the researchers or volunteered. Participants were included if they were 13 years of age or older with a best corrected visual acuity of 20/25 or better between their eyes. The participants consisted of two groups; the intermittent exotropia group (IXT group) had a near exodeviation of more than 10 prism diopters (PD), and the control group were orthotropic or had exophoria of less than 5 PD. Patients with the following conditions were excluded: a distance deviation greater than their near deviation (divergence excess type IXT), a corrected visual acuity worse than 20/25, amblyopia with a difference of more than two lines, paralytic or restrictive exotropia with limitations in duction and version, sensory exotropia where binocular fusion is not possible due to poor visual acuity, a coexisting vertical deviation greater than 5 PD, or dissociated vertical deviation. Ocular diseases, chromosomal abnormalities, or systemic disorders that could affect ocular alignment, such as periocular tumors, chronic progressive external ophthalmoplegia, or myasthenia gravis, were exclusion criteria.

The study protocol complied with the Declaration of Helsinki and was approved by the Institutional Review Board of Seoul National University Bundang Hospital (B-1909-562-303). Informed consent was obtained from all of the subjects after the details of this study were explained.

### 2.2. Physical Examination

Refractive errors were measured through cycloplegic refraction with cyclopentolate hydrochloride 1% eye drops. Participants with myopia greater than −1.00 D were prescribed spectacles with full cycloplegic refraction. Patients with hyperopia greater than +3.00 D were prescribed spectacles approximately +1.00 to +1.50 D less than full cycloplegic refraction. The angle of deviation was measured using the alternate prism cover test at 1/3 m and 6 m. Stereopsis was assessed using the Randot stereotest (Stereo Optical Company, Chicago, IL, USA) and converted into logArcsec equivalents. The near point of accommodation (NPA) was measured while the participants monocularly focused on the “E” one line above their near visual acuity threshold, and the near Snellen E chart was gradually moved toward them until they reported that the letters were blurred and they could no longer maintain a clear image. The near point of convergence (NPC) was measured using the push-up method and an Astron accommodative rule (Gulden Ophthalmics, Elkins Park, PA, USA). The target was gradually moved toward the participants until they could no longer maintain a single image and reported double vision, or the examiner noted ocular divergence. The NPA and NPC were measured three times in centimeters, and the average was recorded [18].

### 2.3. The Eye Tracking Procedure

The eye tracking device (Tobii Technology, Stockholm, Sweden) transmitted gaze data to the computer at 40 Hz intervals. The eye tracking device worked with the display monitor to track the gaze positions. All gaze data were mapped into a 2D coordinate system aligned with the active display area. The left end of the screen was defined as coordinate 0, the right end was defined as 100, and the position of the gaze was expressed in corresponding numbers.

**Figure 1:**
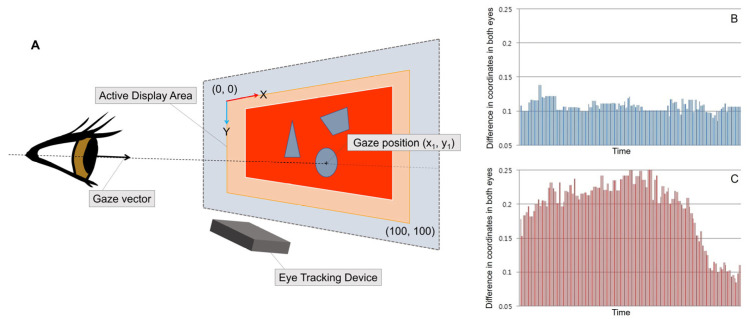
(**A**) Schematic drawing of eye tracking device and gaze tracking results. The eye tracking device transmits gaze data to the computer at 40 Hz intervals. The eye tracking device works with the display monitor to track gaze positions. (**B**) Example of eye tracking data from the control group. The differences between gaze coordinates in both eyes are relatively constant. (**C**) Example of eye tracking data from the intermittent exotropia group. The differences between the gaze coordinates in both eyes are increased during the early deviation period.

The participant was seated in a comfortable position 45 cm in front of the screen to which the eye tracking device was attached (Figure 1). The eye tracking device first detects and calibrates the participant’s head and eye positions. The calibration process consists of the participant positioning their face as guided by the eye tracking device on the screen and binocularly looking at dots that appear in nine locations in sequence. Because the device is intended for consumer applications, there are few technical specifications provided by the manufacturer about how this process calibrates the eye tracking process. Once the eye tracking device recognizes the participant’s position, it allows for free head movement while the user’s head is within 45~80 cm of a virtual tracking box, which has the shape of a frustum, with the vertex positioned in the center of the device [19]. However, to ensure the consistency of the test, the examiner encouraged the participant to remain as still as possible.

After the eye tracking device completed calibration, the participant performed three tasks: (1) horizontal smooth pursuit following the points continuously moved horizontally, (2) vertical smooth pursuit following the points continuously moved vertically, and (3) random dot fixation onto a static dot randomly appearing at one of 9 fixed positions on the screen. The tasks were completed in a total of 14 s, and the average of the data from the two repetitions was recorded.

### 2.4. Automated Fusion Control Assessment

The data acquired by the eye tracking device during each visual task were processed into the following three sequences: (1) The mean difference in the gaze distance between both eyes. (2) The gaze recognition rate, defined as the percentage of times the eye tracking device missed the participant’s gaze. Data from patients with a gaze recognition rate of <50% for a given task were excluded from further evaluation of the validity and reliability of the eye tracking device’s measurements. (3) The fixation disparity (FD) score, defined as the percentage of the number of times the gaze difference between both eyes exceeded the upper limit of the 95% confidence interval of the mean difference in the normal control group.

The control group was necessary for several reasons. Firstly, in practice, orthophoria, which is ideal perfect eye alignment, is very rare, and most people have a small heterophoria and a slight loss of fusion control [20]. Therefore, defining the state of fusion control as an exactly zero difference in position between the two gazes using an eye tracking device was impossible. Instead, it should be defined as the average gaze alignment of normal individuals. Secondly, the eye tracking device was not flawless, as there was some noise during the eye tracking process. This noise can be caused by various factors, such as momentary loss of corneal reflections, reflections from external ocular surfaces, and inaccurate determination of the pupil’s center position [21]. It was therefore necessary to compare the results obtained from eye tracking in normal participants, including the noise.

### 2.5. The Conventional Fusion Control Assessment

The participants’ fusion control scores were measured by a trained ophthalmologist (D.H.K). In this study, the level of control was evaluated using two systems, the NCS and the OCS, and the scoring scheme was implemented according to the method suggested in previous reports [11,12,14,15]. The NCS is a 9-point rating scale for grading the control of IXT, consisting of a subjective home control score and a clinical far/near control score [11,12]. The OCS is a scale that evaluates the far/near control of IXT on a 10-point scale through observation for 30 s, and the mean of triple measures is obtained [13,14,15].

### 2.6. Validity and Reliability

The agreement between the automated fusion control scores measured using the eye tracking device and the conventional fusion control scores were assessed using the Spearman’s rank correlation coefficient. The correlations between the FD score measured using the eye tracking device and the OCS and NCS were analyzed for each task.

The test–retest reliability of the automated fusion control scores measured using the eye tracking device was assessed using the absolute agreement model of the intraclass correlation coefficient (ICC) and the 95% limits of agreement (LoAs), represented in the form of Bland–Altman plots. The ICC indicates the relative reliability and is interpreted using the following criteria: an ICC > 0.75 specifies excellent reliability, and 0.40 < ICC < 0.74 represents fair to good reliability [22].

The data were analyzed using SPSS version 21.0 (IBM SPSS Statistics for Windows, V.21.0; IBM Corp, Armonk, NY, USA). Normality was checked using the Shapiro–Wilk test, continuous variables using Student’s *t* test, and data with a non-normal distribution with the Mann–Whitney U test. The results were interpreted as statistically significant at *p* < 0.05.

## 3. Results

### 3.1. Participant Characteristics

A total of 25 participants were enrolled for automated and conventional fusion control in intermittent exotropia, and 25 control subjects were enrolled to provide normative values. The IXT group consisted of 13 men, while the control group had 10 men. The age of the IXT group ranged from 15.5 to 40.7 years, with a mean of 24.3 ± 6.6 years, while the control group had a mean age of 26.8 ± 2.5 years, ranging from 21.4 to 30.9 years. In the IXT group, the NPC measured 8.7 ± 3.0 cm (range: 3.5 to 26.0 cm), while in the control group, it measured 7.9 ± 2.0 cm (range: 5.0 to 13.0 cm). The NPA measurements were 9.8 ± 3.7 cm (range: 4.8 to 19.0 cm) in the IXT group and 9.1 ± 2.0 cm (range: 5.5 to 14.5 cm) in the control group. Except for stereopsis, all of the characteristics were similar between the two groups. Stereopsis was 1.95 ± 0.29 (range: 1.51 to 2.60) in the IXT group and 1.58 ± 0.30 (range: 1.10 to 2.00) in the control group (*p* < 0.001). (Table 1).

### 3.2. Assessment of Automated and Conventional Fusion Control

The automated assessment of fusion control using the FD score in the IXT group showed a mean value of 23.6 ± 31.0 for horizontal smooth pursuit, 26.0 ± 33.6 for vertical smooth pursuit, and 25.1 ± 32.5 for random dot fixation.

The results of the conventional assessment of fusion control in the IXT group measured using the NCS and OCS are presented in Table 1. The average scores in the NCS and OCS were 4.5 ± 1.7 and 4.1 ± 2.0, respectively (Table 2).

### 3.3. Validity of the Automated Fusion Control Assessment

**Figure 2:**
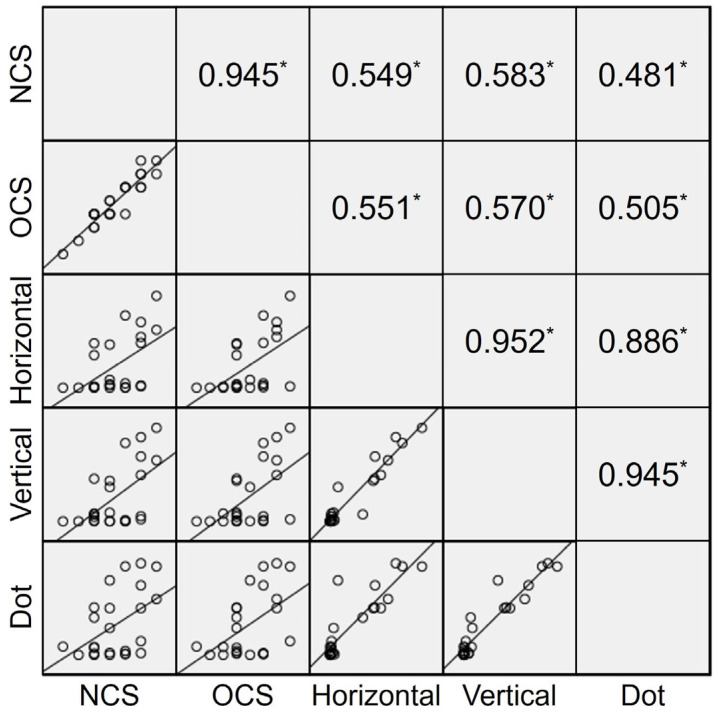
Scatter plot and Spearman’s correlation coefficient between automated and conventional assessment of fusion control. Newcastle Control Score (NCS) and Mayo Clinic office-based scale (OCS) score were positively correlated with the fixation disparity (FD) scores of horizontal pursuit, vertical pursuit, and random dot fixation. The FD scores of each task measured with an eye tracking device showed stronger positive correlations with each other compared to the intertest correlations between the conventional tests and the eye tracking device. The asterisk (*) indicates *p* value smaller than 0.05.

The Spearman’s correlation coefficients between the FD score and the NCS/OCS score were determined for each task (Figure 2). The FD scores during horizontal pursuit, vertical pursuit, and random dot fixation showed positive correlations with the NCS (r = 0.549, 0.583, and 0.481, respectively; *p* = 0.006, 0.003, and 0.024, respectively) and OCS score (r = 0.551, 0.570, and 0.505, respectively; *p* = 0.005, 0.004, and 0.013, respectively). The FD scores of each task measured with the eye tracking device showed strong positive correlations between horizontal and vertical pursuit (r = 0.952, *p* < 0.001), horizontal pursuit and random dot fixation (r = 0.886, *p* < 0.001), and vertical pursuit and random dot fixation (r = 0.945, *p* < 0.001).

The analysis of the near score on the OCS and the FD score showed a stronger positive correlation for all three tasks (Spearman’s correlation analysis, r = 0.683, 0.699, and 0.751, respectively) (Table 3).

### 3.4. Reliability of the Automated Fusion Control Assessment

**Figure 3:**
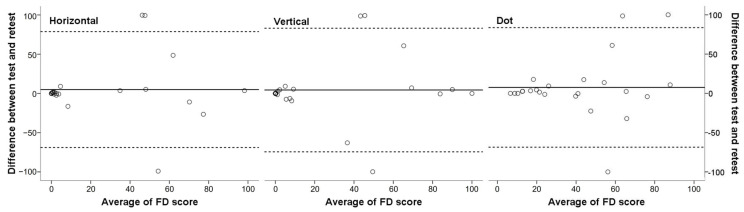
Bland–Altman plots showing test–retest reliability for FD scores measured using the eye tracking device. The solid line shows the mean difference, whereas the dashed line shows 95% limits of agreement. The half-width of the 95% limits of agreement represented for each task were ±73.8 for horizontal pursuit, ±78.8 for vertical pursuit, and ±76.2 for random dot fixation. The test–retest reliability was poorer for exotropia patients with a larger FD score regardless of the type of task.

The test–retest reliability of the automated scores for fusion control measured using the eye tracking device is summarized in Table 4. The reliability of the automated fusion control scores, represented by the FD score and determined using the ICC, was 0.633 for horizontal pursuit, 0.656 for vertical smooth pursuit, and 0.697 for random dot fixation (all *p* values < 0.05 using the ICC). Bland–Altman plots and the 95% limits of agreement were obtained for repeated tests of each visual task (Figure 3). As shown in Figure 3, the half-width of the 95% limits of agreement represented for each task were ±73.8 for horizontal pursuit, ±78.8 for vertical pursuit, and ±76.2 for random dot fixation. Meanwhile, exotropia patients with a large FD score showed poor test–retest reliability regardless of the type of task.

## 4. Discussion

In this study, a newly developed automated system for measuring fusion control of IXT using an eye tracking device was tested and compared to conventional scoring systems. The fixation disparity (FD) scores, defined as the percentage of times the gaze difference between both eyes exceeded the estimated normal range, showed fair to good reliability with the NCS and OCS during all three tasks: horizontal pursuit, vertical pursuit, and fixation on randomly appearing dots. In addition, automated fusional control scores measured using the eye tracking device showed good test–retest reliability in patients with IXT.

Fusion control is an important indicator of the deterioration or improvement of strabismus and a major criterion for applying surgical correction [23]. Observing strabismus for more than 50% of waking hours and poor control on the cover test suggest the need for surgical correction [24]. Chia et al. [25] observed 287 IXT patients for 5 years and reported that the control of exodeviation worsened in about 20% of them. Buck et al. [26] reported that the degree of fusion control correlated with stereoacuity and the angle of deviation, which are traditional indicators of the severity of strabismus. In this study, patients with poor control eventually required surgery [26]. In addition, in several studies, a better degree of control was a predictive factor of a higher surgical success rate [17,27,28]. In the evaluation of fusion control in IXT, many clinicians have used a variety of scoring systems, such as the NCS or the OCS or the Look And Cover, then Ten seconds of Observation Scale for Exotropia (LACTOSE) control scores [11,12,13,14,15,16,17]. However, these tests have inherent drawbacks per se that hinder their widespread adoption in clinical practice. Despite the good agreement between different examiners, the conventional scoring systems rely on the subjective interpretations of the examiner/parents [29]. Therefore, the accuracy of the measurements depends primarily on the expertise of the examiner and the level of cooperation of the participants [14,15].

The eye tracking device has several advantages in measuring the degree of fusion control in intermittent exotropia. First, it is an automated system that enables objective measurement and improvements in consistency. Economides et al. [21] measured how often exodeviation occurs throughout the day using eye tracking glasses and reported that patients and families tended to exaggerate the severity of exotropia compared to the measurements obtained using eye tracking glasses. This suggests that conventional assessments can be inaccurate and that objective measurements are needed, such as eye tracking devices. Second, the use of an eye tracking device does not require one eye to be covered during testing. Instead, both eyes remain open and focus on a single target, minimizing the impact of dissociation on the assessment of strabismus [30]. Third, the eye tracking device is useful for follow-up and comparison by quantifying the loss of fusion control as the FD score. Finally, since this test uses targets displayed on a screen, it can make it easier for children of younger ages to concentrate more effectively and minimize distraction.

In our study, the evaluation of fusion control with the eye tracking device showed similar results to those of the conventional scores regardless of the type of task. The validity of the FD score during horizontal pursuit, vertical pursuit, and random dot fixation showed a positive correlation with the NCS (r = 0.549, 0.583, and 0.481, respectively) and OCS score (r = 0.551, 0.570, and 0.505, respectively). Meanwhile, there was a higher positive correlation between the three tasks performed using the eye tracking device (r = 0.952, 0.886, and 0.945, respectively) compared to the intertest correlation between the eye tracking device and the conventional tests. This difference may be due to the discrepancy in subjective grading scales and objective measurements derived through thresholds compared to normative values.

In our study, the test–retest reliability of measuring fusion control in IXT with the eye tracking device was favorable. However, patients with a larger mean FD score showed poor reliability regardless of the type of task in the Bland–Altman plots. This is consistent with the findings of previous studies, showing greater variability in the control in IXT patients with poor fusion control [15]. Therefore, multiple measurements should be taken to reveal the true state of fusional control in IXT.

There are some limitations that should be considered in this study. First, the correlation coefficient between the FD score and the NCS and OCS score was fair to good. As the working distance of the eye tracking device is 45 cm, our study could only measure the fusion control of IXT during near to intermediate fixation. The results of our study do not reflect the distance control in IXT, which probably contributed to the low–moderate correlations, and further studies in different settings may be needed to clarify this issue. We also differed from the conventional methods in that we assessed both eyes without dissociating them. However, this was unavoidable because there is no other recognized assessment method like the NCS or OCS score that can assess fusion control without dissociating both eyes. Second, our results depended on an estimated normal range derived from an age-matched control group without significant strabismus. We measured the FD score based on the upper limit of the 95% confidence interval of the mean fixation disparity in the control group. However, due to the relatively small number of subjects in our study, future studies with a larger sample size and a normative database would provide more confirmative evidence. Finally, the eye tracking device was utilized without dissociation, which is difficult to apply to IXT with good control, such as scoring 0–2 points in the OCS score, as it is not revealed before dissociation.

In conclusion, the newly developed system for the automated measurement of fusion control may assist in detecting and quantifying fusion control in IXT. As a new system for evaluating the severity of strabismus, comprehensive validity studies are mandatory for it to be efficiently implemented across studies regarding various type of strabismus.

## Figures and Tables

**Table 1:** Comparison of baseline characteristics.

Characteristics	IXT Group (*n* = 25)	Control Group (*n* = 25)	*p* Value
Gender (M:F)	13:12	10:15	0.571 *
Age at examination (y)	24.3 ± 6.6 (15.5~40.7)	26.8 ± 2.5 (21.4~30.9)	0.095 ^†^
Distance best corrected visual acuity (LogMAR)	0.00 ± 0.01 (0.00~0.02)	0.00 ± 0.01 (0.00~0.05)	0.428 ^‡^
Near best corrected visual acuity (LogMAR)	0.00 ± 0.01 (0.00~0.05)	0.00 ± 0.00 (0.00~0.00)	0.317 ^‡^
Spherical equivalent refractive errors (D)	−3.19 ± 2.74 (−9.88~+1.81)	−3.00 ± 2.89 (−8.94~+0.69)	0.814 ^†^
Stereopsis (LogArcsec)	1.95 ± 0.29 (1.51~2.60)	1.58 ± 0.30 (1.10~2.00)	<0.001 ^†^
Near point of convergence (cm)	8.7 ± 3.0 (3.5~26.0)	7.9 ± 2.0 (5.0~13.0)	0.245 ^†^
Near point of accommodation (cm)	9.8 ± 3.7 (4.8~19.0)	9.1 ± 2.0 (5.5~14.5)	0.430 ^†^
Distance deviation (PD)	21.1± 13.0 (4~50)	0.2 ± 0.9 (0~4)	<0.001 ^‡^
Near deviation (PD)	27.1 ± 10.0 (14~50)	0.8 ± 1.4 (0~4)	<0.001 ^‡^

Data are numbers or mean ± standard deviation values (minimum~maximum). IXT = intermittent exotropia; M = male; F = female; y = years; LogMAR = logarithm of the minimum angle of resolution; D = diopters; PD = prism diopters. * *p* value according to the Chi-square test, ^†^ Student’s *t* test, ^‡^ Mann–Whitney U test.

**Table 2:** Automated and conventional assessments of fusion control in patients with intermittent exotropia.

**Automated** **assessment**	**Score**										
Horizontal FD	23.6 ± 31.0 (0–98.20)										
Vertical FD	26.0 ± 33.6 (0–100)										
Dot FD	25.1 ± 32.5 (0–96.70)										
**Conventional** **assessment**	**Score**	0	1	2	3	4	5	6	7	8	9
NCS	4.5 ± 1.7 (1–8)	0	1 (4%)	1 (4%)	6 (24%)	5 (20%)	4 (16%)	5 (20%)	2 (8%)	1 (4%)	0
OCS	4.1 ± 2.0 (1–9)	1 (4%)	1 (4%)	2 (8%)	8 (32%)	2 (8%)	5 (20%)	3 (12%)	2 (8%)	0	1 (4%)

Data are presented as means ± standard deviations (range) and/or numbers (%); FD = fixation disparity (percentage of number of times the gaze difference between both eyes exceeded the upper limit of the 95% confidence interval of the mean difference in the normal control group); NCS = Newcastle Control Score; OCS = Mayo Clinic office-based scale.

**Table 3:** Spearman’s correlation coefficient between automated fusion control score and near control score on Mayo Clinic office-based scale (OCS).

		Horizontal	Vertical	Dot
OCS	Correlation coefficient	0.683	0.699	0.751
	*p* value	<0.001	<0.001	<0.001

**Table 4:** The test–retest reliability of measuring automated fusion control using an eye tracking device.

Score	ICC	95% CI	*p* Value
Horizontal FD	0.633	0.146–0.842	0.011
Vertical FD	0.656	0.197–0.852	0.008
Dot FD	0.697	0.307–0.869	0.003

ICC = intraclass correlation coefficient (Kendall coefficient concordance); CI = confidence interval; FD = fixation disparity (percentage of the number of times the gaze difference between both eyes exceeded the upper limit of the 95% confidence interval of the mean difference in the normal control group).

## Data Availability

The Institutional Review Board of Seoul National University Bundang Hospital/Ethics committee has placed ethical restrictions to protect patient identities. However, the data are available to anyone who is interested without restriction. A minimal data set can be made available upon request. For data requests, please contact the SNUBH IRB office at 82-31-787-8804, 98614@snubh.org.
